# Nephritis from Calculus, Simulating Cancer of the Stomach

**Published:** 1847-11

**Authors:** 


*Nephritis from Calculus, Simulating Cancer of the Stomach.*—A Captain of infantry entered the Hospital of Val-de-Grace, under M. Champouillon. For seven years, this officer, of a sanguine and robust constitution, had complained of pain in the epigastrium, increased during digestion, and attended with vomiting after a tolerably full meal. For these sufferings the patient consulted numerous physicians, all of whom spoke of gastralgia, of chronic gastritis, and consequently prescribed for him repose, baths, and a vegetable and mik diet. This regimen always produced a notable amelioration of the symptoms, so as to give the patient the idea of recovery. However, a new and final relapse occurred at the end of March, and the captain determined to enter the hospital, which he did on the 7th of April. The vomitings were then very painful, and almost continual; the belly was distended; the feet and hands presented a moderate amount of serous infiltration. Leeches were applied to the epigastrium, and ice placed on, and which was also taken internally, with morphia. The vomiting was by these means suspended for a few days, and then returned. The tongue, previously dry and brown, began to be moist and white. The matters vomited, at first white and glairy, became on the 12th of April, of a blackish colour. On a microscopical examination, they were found to be composed of mucus, and of a granular matter, which M. Millon recognized as blood-discs, altered in form. On the 13th, a consultation was held. The physicians present attempted in vain to percuss the epigastric region. The abdomen, always distended, prevented a manual examination, and the slightest pressure also caused the most severe pain. This circumstance, coupled with the nature of the matters ejected, and the straw yellow tint of the countenance, gave rise to the opinion that, instead of chronic gastritis, there was cancer of the stomach; and this opinion was strengthened by the fact, that his mother had suffered from cancer of the womb. Ice and opium were given; but the vomiting persisted till death, which occurred on the 16th. A post mortem examination was made the next day. The mucous membrane of the stomach was found slightly injected towards the great cul de sac, but appeared elsewhere quite healthy. The intestines, liver, and spleen, presented no pathological lesions. The kidneys were very large, imbedded in a layer of compact cellular tissue, to which they were firmly adherent. The cortical substance, considerably atrophied, was of a decidedly black colour. Each kidney enclosed a calculus of lithate of ammonia, rugose and mamillated, and of the size of a pigeon's egg. Several smaller calculi were contained in the pelves of the kidneys.

Here, then, was a complete error in diagnosis. The symptoms were such as might, with the greatest probability, be referred to cancerous disease of the stomach, with ulceration. In forming the diagnosis, no aid could be drawn from a manual examination of the epigastric region, and from palpation; but the same thing may happen in cancer of the stomach. M. Champouillon, indeed, in making his examination, and pressing on the lateral and posterior region of the abdomen, had excited acute pain. This pain, coinciding with the œdema of the extremities, and the frequent vomiting, at once led him to suspect nephritis from calculus, or with albumen. Consequently, he examined the urine of the patient to detect albumen, or an excess of lithic acid; but the re-agents employed gave no indications of either; and moreover, the patient never having complained of pain in the lumbar region, or of any derangement in the functions of the kidney, the idea of renal disease was given up.—*Dub. Med. Press, from the London Lancet.*

